# ‘Your country needs you’: the ethics of allocating staff to high-risk clinical roles in the management of patients with COVID-19

**DOI:** 10.1136/medethics-2020-106284

**Published:** 2020-05-14

**Authors:** Michael Dunn, Mark Sheehan, Joshua Hordern, Helen Lynne Turnham, Dominic Wilkinson

**Affiliations:** 1 The Ethox Centre and Wellcome Centre for Ethics and Humanities, University of Oxford, Oxford, UK; 2 Faculty of Theology and Religion, University of Oxford, Oxford, UK; 3 Oxford University Hospitals NHS Foundation Trust, John Radcliffe Hospital, Oxford, UK; 4 Oxford Uehiro Centre for Practical Ethics, University of Oxford, Oxford, UK; 5 Murdoch Children’s Research Institute, University of Melbourne, Melbourne, South Australia, Australia

**Keywords:** clinical ethics, professional - professional relationship, health personnel, health care for specific diseases/groups

## Abstract

As the COVID-19 pandemic impacts on health service delivery, health providers are modifying care pathways and staffing models in ways that require health professionals to be reallocated to work in critical care settings. Many of the roles that staff are being allocated to in the intensive care unit and emergency department pose additional risks to themselves, and new policies for staff reallocation are causing distress and uncertainty to the professionals concerned. In this paper, we analyse a range of ethical issues associated with changes to staff allocation processes in the face of COVID-19. In line with a dominant view in the medical ethics literature, we claim, first, that no individual health professional has a specific, positive obligation to treat a patient when doing so places that professional at risk of harm, and so there is a clear ethical tension in any reallocation process in this context. Next, we argue that the changing asymmetries of health needs in hospitals means that careful consideration needs to be given to a stepwise process for deallocating staff from their usual duties. We conclude by considering how a justifiable process of reallocating professionals to high-risk clinical roles should be configured once those who are ‘fit for reallocation’ have been identified. We claim that this process needs to attend to three questions that we consider in detail: (1) how the choice to make reallocation decisions is made, (2) what justifiable models for reallocation might look like and (3) what is owed to those who are reallocated.

## Introduction

The COVID-19 pandemic is placing increasing demand on health services, particularly in critical care. This has required healthcare providers to change well-established care pathways and staffing models. Specialised roles are being created, including the development of ‘intubating teams’.^1^ These are teams made up of anaesthetists, anaesthetic nurses and operating department practitioners whose primary role is to provide safe intubation to critically ill patients able to benefit from intensive care therapy. More broadly, staff members are being reallocated from clinical specialities in other parts of the health service to acute care settings such as the intensive care unit or the emergency department. Similar reallocation strategies are also being invoked in community health and care settings.

These health and care professionals are being asked to provide care, or to deliver specific interventions, in environments that pose new and significant risks to themselves. There is particular concern that these professionals are at increased risk of acquiring COVID-19 themselves. This is because the airway secretions of patients who become seriously ill with COVID-19 contain the highest concentration of virus particles, and therefore, clinical interventions that expose healthcare workers to these secretions are particularly dangerous. These include interventions, such as intubation, that create aerosols of virus-laden secretions.^2^ There is anecdotal evidence that COVID-19 has qualities of dose-dependency and therefore those undertaking these high-risk interventions might develop more severe illness.^3^

As members of clinical ethics advisory groups supporting hospitals at this time, we have encountered considerable uncertainty and worry among health professionals about whether and how they and their peers should be allocated to high-risk clinical roles. In this paper, we seek to make progress in analysing this issue.

## Duties of care and duties to treat: initial ethical considerations

Practical questions of staff allocation might be seen as ethically straightforward if a simplistic account of health professionals’ obligations towards patients is endorsed. This account might go something like this: doctors have well-established professional duties towards patients by virtue of their social roles, and these duties extend to what is sometimes described as a ‘special positive duty’ to treat patients when they possess the relevant skills. Such an account looks consistent with recent guidance produced in the UK, which states that ‘Doctors are bound by their duty of care for patients in the pandemic… To uphold this duty of care, doctors will need to be flexible, and may need to work in locations or clinical areas outside their usual practice’.^4^ If doctors have a strong duty to treat, then any proposed means of allocating staff that maximises benefit for patients might be justified.

The key question here is whether health professionals’ ‘obligation to treat’ is sufficient to ground a requirement to discharge this duty in a pandemic scenario where doing so poses a high risk of harm to the professionals concerned. In line with other commentators, our view is that there are good reasons to conclude that health professionals’ duties do not necessarily extend to an overarching and compulsory requirement to treat patients in such circumstances.

Writing in response to the previous SARS epidemic, Malm *et al* 5 identify compelling counterarguments against a number of claims that attempt to ground the ‘obligation to treat’. Such claims include the provision of explicit or implied consent, the social contract that health professionals have entered into and the existence of professional oaths or codes. Their arguments are that, in the face of personal risk, the ‘obligation to treat’ position does not outweigh the duties professionals have to themselves and their families.

If it is correct that professionals do not have an all-things-considered duty to provide treatment to patients with SARS, then whether this claim applies to COVID-19 will depend in part on how the risk compares. Since the risk for health professionals from COVID-19 appears to vary with different roles, this might mean that professionals could opt out of some duties in this pandemic (performing interventions associated with increased risks) but not others (providing ancillary care, comfort care, palliative care and non-risky interventions to patients).

One obvious objection to the view that health professionals do not have a ‘duty to treat’ in this context is that it must also apply also to those professionals who are not being redeployed but who are facing increased risks in the performance of their usual clinical duties. Could those working in the intensive care unit (ICU), for example, opt out of working in this setting during the pandemic on this basis?

The ‘implied consent-based’ reasons might be stronger here since those who regularly work in the ICU could reasonably expect their usual clinical activities to give rise to some additional risks. However, it is unlikely that ICU staff, for example, have given explicit or implicit informed permission to take on greater than normal risks. Furthermore, such consent, even if given, would not usually be taken to be binding. Professionals might revoke their prior consent and now decline to fulfil these tasks. Our conclusion here is that the duty to treat arguments, whatever force they have, apply both to those now working in higher risk areas and to those being redeployed.

Accepting the position advanced by Malm and colleagues, and developed in this way, produces an interesting tension between the existence of an overarching obligation on the health service to meet patients’ needs and the persuasive view that an individual health professional’s duties do not extend to treating patients when doing so would place them at heightened risk of harm. This essentially is the ethical bind that lies at the heart of the issue: pandemic treatment needs to be provided, but no individual in a healthcare role is specifically obliged to provide it.

## Addressing new asymmetries in health care need: phases of deallocation and reallocation

The underpinning rationale for staff reallocation is that healthcare depends on having professionals with the appropriate skills in the right place at the right time. The aim is to ensure the best outcomes for all patients. The COVID-19 pandemic is leading to increasing asymmetries of need across the whole health service, and reallocating clinical staff becomes an important responsive strategy, when staffing levels are finite, to address this issue.

It makes sense to distinguish three distinct steps in any process of redeploying staff from one part of the healthcare system to another:

1. An overall assessment of the nature of the need and the asymmetry.
2. A deallocation or deprioritisation process in which staff are freed from their established roles and responsibilities.
3. A redeployment process.

In what follows further, we are mostly concerned with the third step, but it is important to articulate how the first two steps ought to be managed.

### Overall assessment of provision

The contexts we are considering here are those where there is an asymmetry of provision and so, presumptively, a newly emerging and ethically problematic inequality in care for patients overall. It is important that the need for redeployment and the benefit achieved/harm avoided by redeployment is articulated clearly and that those involved understand the purposes of these changes. This means explicitly considering the losses to non-COVID-19 patients.

### Deallocating staff from established roles

Some staff will need to be removed from their current positions. This requires a prioritisation exercise in the ‘home’ area. This is ethically significant because it is very likely to affect the care of patients in ways that are problematic: if there are fewer staff in oncology, then oncology patients are likely to suffer. We propose the following three bands to approach the deallocation process.

- Band 1: urgent. The group of patients for which there are significant and avoidable harms associated with delaying treatment.
- Band 2: low priority. The group of patients for which (1) there are no, or limited, harms associated with delaying treatment and (2) the group of patients for which there is very low chance of treatment being effective.
- Band 3: mitigation. The group of patients for whom there are measures that can be put in place to mitigate the harms associated with delaying treatment.

These bands are intended to help with general allocation of staff as well as specific cases and case load management in the context of a pandemic. For general staffing, the number and specialisation of staff should be such that: (1) urgent cases can continue to be managed, (2) mitigation measures are established and (3) basic level care, including comfort and palliative care, can be provided, in line with the ethical principle of non-abandonment.^6^

Each of these bands should be considered temporally. For example, it might be required to assess whether there would be ‘significant and avoidable harm involved in delaying treatment for 2 weeks’. The precise timeframe should be determined on the basis of the general resource pressure and also according to what is practical in the context. Equally, as resources are stretched, the level of harm able to be accommodated in the urgent band will go up (and vice versa). In extreme resource scarcity, difficult decisions made about the provision of even basic level care might be required.

Patients should be reassured that the judgements about these bands will be revisited and that there is likely to be movement between bands, depending on context. Transparency here is important.

## Processes and models for reallocating health professionals

Once an overall strategy of deallocation has been formulated, the question of reallocation should be attended to. Here, an additional set of issues arises: what approach should be adopted to determine which health professionals who are identified as being ‘fit for reallocation’ are chosen for redeployment?

There are three subsidiary questions here: (1) what constitutes a justifiable process for deciding which staff are chosen to be reallocated, (2) what reallocation models for making these choices are justifiable and (3) what is owed to those reallocated to high-risk clinical roles?

### What would constitute an appropriate process to make decisions about staff reallocation?

Addressing the first question requires an appropriate procedure for making a choice about reallocation to be determined. There are three broad options for determining how such choices could be made:

*Option 1: no choice*. The first option is for those who are organising or coordinating healthcare delivery (perhaps administrators or clinical leaders in a hospital) to redeploy staff in a way that best addresses the new asymmetry of need. Adopting this option requires staff to be conscripted into roles, irrespective of their preference. Health professionals could, in principle at least, be unable to opt out of being reallocated.

This approach is likely to be efficient, particularly given the need to make decisions rapidly. It may also be the most informed approach, since those at higher levels will potentially have most knowledge of the relevant logistics and clinical demand. However, such an approach will be manifestly unfair and, arguably, unethical: it would allow no staff member to retain control over how their job is configured and enacted, nor potentially to uphold other duties that they possess beyond those connected to their working lives.

*Option 2: individual choice*. The second option accords the choice about reallocation to each individual professional. On this option, professionals are able to volunteer, or not, on the basis of their individual preferences. The pros and cons of this approach to making a choice are considered below, in the ‘volunteering model’ of redeployment.

*Option 3: collective choice*. The third option comprises a series of potential strategies of choosing that are connected by the fact that they accord some decision-making authority to those in the relevant group of professionals who are ‘fit for reallocation’. This option encapsulates a range of decision-making frameworks for collective choice. Such frameworks would include deliberative, consensus-building decisions or a consultation approach in which the final decision is shaped outside of the collective by an administrator or clinical leader.

There are two main arguments in favour of collective choice. First, giving control over the reallocation process to those who are in the frame to be reallocated is likely to ensure that well-established and productive team dynamics are not disrupted (although it is possible that, if no consensus can be reached, this reality might produce new and problematic dynamics of its own). Second, this approach also preserves individuals’ moral agency. The relevant professionals get to decide the basis on which they ought to discharge their responsibilities as a team that recognises their shared responsibilities to act in the interests of patients when there is no one else who can fulfil this task.

Concerns might, however, arise. A participatory model of decision making might be easiest and most practical with small groups of professionals, and it may be challenging or impossible with large or disparate groups to reach a meaningful consensus in a short period of time. Moreover, an emerging consensus might be forged in a way that largely replicates existing and potentially toxic power dynamics within a group, for example, between doctors, nurses and allied health professionals or between grades within any one such cohort.

Whether it is feasible or appropriate to involve professionals themselves in determining decisions about reallocation, it is also important to consider different reallocation models. We think that there are three main models worthy of consideration. Of course, if the choice rests in the hands of the community of professionals, these professionals might diverge from these models in the process of consultation or building consensus. They might also decide that aspects of these different models should be combined or that additional components (such as opt-out procedure) should be invoked. Notwithstanding this, considering viable models provides a useful starting point for the strategies that a collective decision-making process might consider.

### What staff reallocation models are justifiable?

#### Volunteering

The volunteering model adopts an ‘opt-in’ process to select staff for redeployment. Potential volunteers would need to be provided with full information about the roles that they would be adopting, including information about the likelihood of the raised risks involved. It would also be important to explain the background rationale for redeployment to potential volunteers, outlining that redeployment is necessary to meet the health service’s basic duty to meet all patients’ health needs. In so doing, volunteers will be in a position to make a decision in line with their personal assessment of the ethical trade-off to be made. Candidate volunteers are likely to have different motivations (altruistic or non-altruistic) for volunteering; these motivations should not necessarily have implications for whether an individual is permitted to volunteer.

Enabling volunteers to come forward recognises individuals’ understanding of their own professional identity or sense of vocation and the sense of obligation that many individuals will freely accept pursuant to that identity or sense of vocation. Not only then will people remain in full control of their actions within their professional roles, but they will also have an opportunity to freely realise an important part of their vocation at this pivotal moment in their own professional and personal lives.

The viability of the volunteering model will hinge on local characteristics. It might not be possible to recruit sufficient volunteers, particularly if people are being asked to step into specialised roles drawn from a small cohort of qualified personnel. There might also be justifiable limits placed on those eligible to volunteer. Some potential volunteers might be at higher than average risk of harm through exposure to COVID-19 once reallocated. This might include, for example, pregnant women (where there is current uncertainty about the risks to the fetus), staff from black and minority ethnic backgrounds (where there is current uncertainty about the heightened risk that these groups face), staff with pre-existing health problems, older age or compromised immune systems and, potentially, staff identified as being of particular risk of stress or burnout. An overall judgement needs to be made about whether the healthcare system’s obligation to ensure that professionals are protected in the performance of their job outweighs the benefit to patients in them taking on this role. If so, it would be justifiable to prevent these people from volunteering.

Preventing people from volunteering might look unreasonably paternalistic, since we usually allow people to make decisions in their everyday life that are associated with significant risk. Indeed, even in their professional lives, doctors are permitted to volunteer for risky roles in overseas humanitarian organisations, for example. However, if a volunteer were to be at elevated risk of critical illness, and then to become critically ill, this would consume limited resources and reduce staffing levels further. This is also likely to justify a rule that excludes some individuals from volunteering.

Even if it is possible to achieve a sufficient pool of (lower risk) volunteers to meet clinical demand, there is a further question about fairness and the distribution of risk. For example, imagine that out of a pool of 100 eligible anaesthetic staff, 50 volunteers are to be deployed in intubation teams. In that situation, assuming that risk correlates with exposure, those 50 volunteers will be exposed to an additional risk of infection that is two times the risk that they would have faced if the task had been divided evenly among the whole team. Furthermore, those not opting-in will face no additional risk and might be thought to be freeriding on the good will of their colleagues. So, while the volunteering model does allow individuals greater control over the risks that they are exposed to, it will permit potential unfairness in how exposures to those risks are configured.

#### Lottery

An alternative (or indeed necessary addition) to the volunteering model, and one which directly attends to that model’s intrinsic unfairness, is to randomly allocate staff for redeployment. Random allocation is arguably the fairest way to fulfil this requirement as it gives everyone an equal chance of being exposed to additional risk. Of course, this approach leaves the decision entirely to chance and thereby does not enable staff members to retain control over the configuration of their working lives or to realise their sense of professional identity in practice.

The lottery might be configured such that people can be allocated to different kinds of roles, depending on how clinical needs arose locally. There could, for example, be a lottery for redeployment to high-risk frontline acute care roles, and one for redeployment to roles that pose lower risks. Eligibility for entry into these different lotteries could be configured on the basis of background skills, as well as pre-existing conditions that render the person more likely to come to harm. Equally, as in provisions that often characterise a draft into military service, a permission for conscientious refusal to be entered into the lottery could be built into the system. This would allow for individuals to provide reasons why they should not be included, whether clinical or personal, which should be attended to compassionately.

#### Equal sharing of risk

A final model of distribution endeavours to share the risks equally between all, rather than randomly allocating staff. On this model, everyone participates, and a rota might be set up that ensures that each eligible member of staff is broadly exposed to the same amount of risk. This model, like the random allocation model, removes individual control and specific choice in favour of fairly sharing the risks.

The key difference between equal sharing and random allocation is how we handle our knowledge of the risks and where they are likely to be incurred. In a setting where we have good reason to think that the risks to staff are higher when intubating patients with COVID-19, say, it makes sense to use this knowledge to improve risk sharing in the distribution process.

Adopting this model ensures that those redeployed into high-risk roles do not face inequitable exposure to risk. Roles should be devised and scheduled in such a way that those who are allocated share the responsibility (and risk) equally between themselves. Such roles would also need to be flexible in order to ensure that all professionals are maximally enabled to discharge their additional responsibilities, including those relating to childcare or other caregiving activities. It would also be unjustifiable for one person to be allocated such that they undertake more intubations than others or for that person to be positioned such that they consistently undertake more high-risk interventions.

### What is owed to those allocated to high-risk roles?

Once an ethically appropriate model has been adopted, it is necessary to consider whether anything special is owed to those professionals redeployed to high-risk roles. These roles are being configured in ways that do not adhere to standard and agreed responsibilities and entitlements: people are being tasked with providing care that is risky to themselves.

#### Appropriate acknowledgement

Regardless of which model is adhered to, the high risks associated with redeployment give rise to a duty on the health service to provide appropriate acknowledgement of those facing these risks. Depending on what is practically feasible, this could comprise an additional financial payment or professional recognition of some kind. Those staff members from outside the UK who work in the health service on temporary employment visas, and who are redeployed in these ways, could have their immigration status, and that of their families, changed such that they are given ‘indefinite leave to remain’ in the UK.

Importantly, these kinds of acknowledgement would function to recognise the special demands of these roles. Therefore, such acknowledgements are not being instigated as incentives to encourage participation in the volunteering model, nor can they be opted out of in any decision-making process for determining how reallocations occur, including a collective one (though, of course, any particular individual might decline acceptance of this acknowledgement). Thus, even if an appropriate acknowledgement has the de facto effect of incentivising people who might otherwise not volunteer to be redeployed, this would not undermine their justification within that model of reallocation.

#### Sufficient preparation

Those redeployed to high-risk roles need to be appropriately prepared to undertake their responsibilities in these roles. They ought to be given the necessary training and support to be able to work to the requisite level and inducted into established clinical teams. Additionally, limited stocks of personal protective equipment need also to be allocated to those redeployed into high-risk roles.

#### Prioritisation for resources

There has been discussion about whether healthcare professionals should be prioritised for access to the ICU or ventilation if they acquire COVID-19 and require critical care, when they are judged to be at equal clinical need as a non-health professional when there are insufficient resources for all patients.^7^ If the arguments that underpin this claim are taken to be convincing, then the health system might also elect to guarantee additional or equal high priority to those who are being redeployed.

It is also arguable that, at a national level, priority provision for compensation should be made for all those being reallocated to high-risk roles, whether on a voluntary basis or not, in the event of their death or of long-term negative health consequences associated with becoming seriously ill with COVID-19.

## Conclusion

The allocation of health professionals to high-risk clinical roles raises distinctive ethical uncertainties. While there is an overarching requirement for health services to meet the needs of patients with severe illness, there is no specific obligation that falls on any individual health professional to provide treatment. Equally, there are tensions between ensuring that staff are allocated to meet needs and ensuring a decision-making process that most fairly and ethically distributes the exposure to risks arising through reallocation.

In this paper, we have sought to clarify the steps that need to be considered in any defensible process of allocating staff to high-risk clinical roles in the face of the COVID-19 pandemic. Precisely how these steps are attended to in practice will be dependent on context: the size of the workforce, the specific manifestation of patients’ needs across a health service and the ways in which roles, responsibilities and working relationships are configured. Having said that, a justifiable process of allocating staff in context will need to attend carefully to distinctive stages of decision making: first, to decisions about both deallocation and reallocation, and second, to decisions about how the process of reallocation is determined and what specific models of reallocation are adopted in this process.
